# Techniques in MRI–Negative Cushing disease

**DOI:** 10.3171/2023.4.FOCVID2340

**Published:** 2023-07-01

**Authors:** Jamie J. Van Gompel, Maria Peris Celda, Matteo Zoli, Juan Fernandez-Miranda

**Affiliations:** 1 Department of Neurosurgery and Otolaryngology, Mayo Clinic, Rochester, Minnesota;; 2 Department of Neurosurgery, Alma Mater Studiorum–Università di Bologna, Bologna, Italy; and; 3 Department of Neurosurgery, Stanford University, Palo Alto, California

Magnetic resonance imaging (MRI)–negative Cushing disease is one of the most difficult and challenging surgical cases in both pituitary and skull base surgery. Experience is gained by a sequence of success and, unfortunately, failure, which results in a hard-fought experience with these cases. There are a variety of established and novel techniques that would benefit from high-quality video documentation, which could help established surgeons and novices alike improve patient care for this difficult population. In this *Neurosurgical Focus: Video* edition, we present high-quality videos addressing pituitary exploration, cavernous sinus exploration with medial wall removal, and methods of subtotal gland resection. In addition, a video correlating the novel imaging technique of intraoperative ultrasound with a prior MRI–Negative case is presented. Finally, a very interesting series of cases demonstrating suprasellar/infundibular disease is presented. We believe this collection of videos will be a valuable resource for the neurosurgery and endocrine community in these most difficult cases, MRI–Negative Cushing disease.

## Disclosures

Dr. Fernandez-Miranda reported personal fees from Stryker, KLS Martin, and Hotry during the conduct of the study.

